# Small vessel cerebrovascular disease is associated with cognition in prospective Alzheimer’s clinical trial participants

**DOI:** 10.1186/s13195-024-01395-x

**Published:** 2024-02-02

**Authors:** Clarissa D. Morales, Dejania Cotton-Samuel, Patrick J. Lao, Julia F. Chang, Jeffrey D. Pyne, Mohamad J. Alshikho, Rafael V. Lippert, Kelsang Bista, Christiane Hale, Natalie C. Edwards, Kay C. Igwe, Kacie Deters, Molly E. Zimmerman, Adam M. Brickman

**Affiliations:** 1 Taub Institute for Research on Alzheimer’s Disease and the Aging Brain, Gertrude H. Sergievsky Center, Department of Neurology, Vagelos College of Physicians and Surgeons, Columbia University, New York, NY 10032 USA; 2 Department of Integrative Biology & Physiology, University of California, Los Angeles, CA USA; 3 Department of Psychology, Fordham University, Bronx, NY USA

**Keywords:** White matter hyperintensities, Amyloid, Alzheimer’s disease, Clinical trial

## Abstract

**Background:**

Secondary prevention clinical trials for Alzheimer’s disease (AD) target amyloid accumulation in asymptomatic, amyloid-positive individuals, but it is unclear to what extent other pathophysiological processes, such as small vessel cerebrovascular disease, account for participant performance on the primary cognitive outcomes in those trials. White matter hyperintensities are areas of increased signal on T2-weighted magnetic resonance imaging (MRI) that reflect small vessel cerebrovascular disease. They are associated with cognitive functioning in older adults and with clinical presentation and course of AD, particularly when distributed in posterior brain regions. The purpose of this study was to examine to what degree regional WMH volume is associated with performance on the primary cognitive outcome measure in the Anti-Amyloid Treatment in Asymptomatic Alzheimer’s Disease (A4) study, a secondary prevention trial.

**Methods:**

Data from 1791 participants (59.5% women, mean age (SD) 71.6 (4.74)) in the A4 study and the Longitudinal Evaluation of Amyloid Risk and Neurodegeneration (LEARN) companion study at the screening visit were used to quantify WMH volumes on T2-weighted fluid-attenuated inversion recovery (FLAIR) MR images. Cognition was assessed with the preclinical Alzheimer cognitive composite (PACC). We tested the association of total and regional WMH volumes with PACC performance, adjusting for age, education, and amyloid positivity status, with general linear models. We also considered interactions between WMH and amyloid positivity status.

**Results:**

Increased frontal and parietal lobe WMH volume was associated with poorer performance on the PACC. While amyloid positivity was also associated with lower cognitive test scores, WMH volumes did not interact with amyloid positivity status.

**Conclusion:**

These results highlight the potential of small vessel cerebrovascular disease to drive AD-related cognitive profiles. Measures of small vessel cerebrovascular disease should be considered when evaluating outcome in trials, both as potential effect modifiers and as a possible target for intervention or prevention.

## Background

Pathogenic models of Alzheimer’s disease (AD) emphasize a biological cascade that begins with amyloid accumulation, followed by tau pathology, neurodegeneration, and subsequent cognitive decline and dementia [1, 2]. While this “amyloid cascade hypothesis” remains quite controversial, it has defined both diagnostic frameworks [3] and strategies for disease treatment and prevention [4]. Observational research from individuals with autosomal dominant, fully penetrant, mutations for AD suggests that amyloid accumulation initiates years, perhaps decades, prior to the onset of clinical symptoms [5]. With the development of neuroimaging and fluidic diagnostic biomarkers for AD, it is now possible to characterize AD pathophysiology in these early, presymptomatic phases of the disease.

Monoclonal antibodies that target the accumulation of amyloid protein remain the primary experimental approach towards disease treatment. Two distinct views have resulted from several clinical antibody trials that failed to show clinical efficacy or modest effects despite evidence of target engagement [6]. The first argues that amyloid pathology is not linked causally to the development and progression of clinical symptoms in AD and questions the fundamental basis of the amyloid cascade hypothesis [7]. The second argues that amyloid removal is still a viable treatment for AD but will only be effective in very early stages of disease pathogenesis [8]. Indeed, among myriad failed trials, three phase 3 trials that included patients with very mild or presymptomatic disease suggest some degree of clinical efficacy in primary [9, 10] or post hoc [11] analyses, which led to FDA approval of lecanemab and aducanumab. Despite the unambiguous ability to clear amyloid from the brain based on changes in biomarker profiles, these medications provide only the mildest clinical benefit to patients, suggesting that amyloid may be one of the factors implicated in AD pathophysiology, but its removal is not a panacea.

Most treatment or secondary prevention clinical trials for AD that target beta-amyloid require that participants have pathophysiological evidence of AD via positron emission tomography (PET) or cerebrospinal fluid biomarkers and have exclusion criteria for significant comorbidities, including suspected cerebrovascular disease. Although there are often other practical or methodological justifications for excluding one pathology in a trial to study another, the primary argument is that in order to determine whether a medication is helpful to individuals suffering from or at risk for a disease, only those with the “purest” forms should be included in trials to ensure that the potential therapeutic outcome of target engagement is not confounded or masked by another disease process. This approach has likely contributed to systematic exclusion of minoritized groups from clinical trials for AD because of differential base rates in comorbidities [12] and otherwise might not be a valid strategy towards evaluating treatment efficacy. Indeed, pathological and neuroimaging data confirm that the majority of symptomatic individuals with AD have evidence of cerebrovascular disease, which cerebrovascular disease precedes symptom onset in AD even in autosomal dominant forms, and that cerebrovascular disease contributes to symptom onset and progression [13–18]. Taken together, the evidence suggests that cerebrovascular disease is a core feature of AD, and imposing exclusion criteria in clinical trials that leave out individuals with suspected or peripheral risk factors for cerebrovascular disease may attenuate but not eliminate its impact on clinical outcomes.

In the current study, we tested the hypothesis that small vessel cerebrovascular disease, operationally defined as white matter hyperintensity (WMH) volume on T2-weighted magnetic resonance imaging (MRI), contributes to primary cognitive outcome performance in a secondary prevention trial for AD. We used screening data from the Anti-Amyloid Treatment in Asymptomatic Alzheimer’s (A4) study, a secondary prevention trial of solanezumab in preclinical AD, and the Longitudinal Evaluation of Amyloid Risk and Neurodegeneration (LEARN) companion study. A unique aspect of A4 and LEARN is that individuals with vascular risk factors were not systematically excluded from participation, allowing us to examine the role of a range of cerebrovascular disease severity in typical prospective AD trial participants.

## Methods

### Participants

Data for the current analyses came from participants who were included in the screening visit for the A4 study, a multicenter clinical trial enrolling cognitively unimpaired older adults (ages 65 to 85) with evidence of increased amyloid accumulation in the brain [8, 19]. This secondary prevention trial evaluated the impact of solanezumab, a monoclonal antibody targeting beta amyloid’s mid-peptide domain, on cognitive progression in individuals characterized as having preclinical AD [19]. Major inclusion criteria for participants in the A4 study were the evidence of elevated brain amyloid levels determined by amyloid PET imaging, the classification of cognitively unimpaired at study enrollment, and access to a study partner willing to participate and provide information on participants’ daily life cognitive function. Major exclusion criteria included receiving treatment for AD with acetylcholinesterase inhibitors; serious or unstable medical, psychiatric, or neurological conditions; suicidality; or recent history of alcohol or substance abuse or dependence [8]. The screening procedure involved an initial cognitive assessment followed by PET imaging to determine amyloid status. After amyloid status was determined, participants with elevated amyloid received structural MRI. Participants determined to be amyloid negative were referred to the Longitudinal Evaluation of Amyloid Risk and Neurodegeneration (LEARN) study [8]. The LEARN study was run in parallel with the A4 clinical trial to act as a comparison group and further characterize preclinical AD vis-à-vis normal aging. LEARN participants received the same longitudinal imaging and clinical assessments as participants in the A4 clinical trial. Imaging data for a total of 1791 screening participants were included in these analyses; Table 1 displays the demographic characteristics of these participants. The enrollment outcome for these participants was not known at the time of our analysis, but all participants underwent cognitive assessment, amyloid PET imaging, and MRI scanning and met inclusion criteria for either the A4 or LEARN studies.

**Table 1:** Demographic features of participants included in the current study

	**A4** **(elevated amyloid)**	**LEARN** **(non-elevated amyloid)**	**Total**	**Test statistic**
***n***	1250	541	1791	––
**Sex/gender**				
Women, *n* (%)	735 (58.5%)	331 (61.2%)	1066 (59.5%)	*χ*^2^ = 0.79 *p* = 0.37
**Age**				
Mean (SD), years	72.0 (4.85)	70.5 (4.30)	71.6 (4.74)	*t* = 6.63 *p* < 0.001
**Ethnicity**				
Hispanic or Latinx, *n* (%)	36 (2.9%)	18 (3.3%)	54 (3.0%)	*χ*^2^ = 0.45 *p* = 0.79
Not Hispanic or Latinx, *n* (%)	1202 (96.2%)	519 (95.9%)	1721 (96.1%)	
Unknown, *n* (%)	12 (1.0%)	4 (0.7%)	16 (0.9%)	
**Race**				
American Indian or Alaskan Native, *n* (%)	2 (0.2%)	5 (0.9%)	7 (0.4%)	*χ*^2^ = 5.75 *p* = 0.21
Asian, *n* (%)	27 (2.2%)	12 (2.2%)	39 (2.2%)	
Black or African American, *n* (%)	32 (2.6%)	14 (2.6%)	46 (2.6%)	
White, *n* (%)	1173 (93.8%)	504 (93.2%)	1677 (93.6%)	
Unknown, *n* (%)	16 (1.3%)	6 (1.1%)	22 (1.2%)	
**Education**				
Mean (SD), years	16.6 (2.81)	16.8 (2.63)	16.6 (2.76	*t* = 1.64 *p* = 0.10
**WMH volume**				
Total, mean (SD), cm^3^	12.27 (9.47)	11.91 (9.05)	12.16 (9.35)	*t* = 0.78 *p* = 0.43
Frontal lobe, mean (SD), cm^3^	4.76 (4.92)	4.47 (4.52)	4.67 (4.81)	*t* = 1.23 *p* = 0.21
Temporal lobe, mean (SD), cm^3^	1.25 (1.17)	1.38 (1.21)	1.29 (1.18)	*t* = 2.00 *p* = 0.04
Parietal lobe, mean (SD), cm^3^	2.67 (3.11)	2.53 (2.91)	2.63 (3.05)	*t* = 0.93 *p* = 0.35
Occipital lobe, mean (SD), cm^3^	2.29 (1.48)	2.14 (1.48)	2.25 (1.48)	*t* = 2.01 *p* = 0.04

### Cognitive assessment

Cognitive testing took place before PET imaging eligibility was determined. The primary cognitive outcome measure used in the A4 study is the preclinical Alzheimer cognitive composite (PACC) [20]. The PACC is derived from four cognitive measures: Free and Cued Selective Reminding Test [21], Logical Memory Test delayed recall (LMDR-IIa) [22], Digit Symbol Substitution Test [23], and Mini-mental State Examination (MMSE) [24]. Scores from each of the four components were normalized using the mean and standard deviation of the sample group; the standardized z scores were then summed to give the composite score used in these analyses [20]. Participants scoring very high (1.5 SD above the norm) or very low (1.5 SD below the norm) on LMDR-IIa were excluded after the first screening visit, before imaging data were collected, in hopes of increasing the likelihood of enrolling participants with a higher risk of imminent cognitive decline associated with AD pathophysiology and removing those with mild cognitive impairment (MCI) [8]. Participants with MMSE scores of 25 to 30, LMDR-IIa scores of 6 to 18, and a Clinical Dementia Ratings of 0 were eligible for further screening with an amyloid PET scan.

### Amyloid PET imaging

Florbetapir PET imaging was used to assess amyloid burden. Images were acquired 50 to 70 min post-injection of 10 mCi of florbetapir F18 and analyzed using a mean cortical standardized uptake value ratio (SUVR) calculated with the whole cerebellum as a reference region [25]. A subset of participants’ amyloid eligibility assessments was verified by two-reader visual consensus before the decision to implement automatic inclusion of participants meeting a composite SUVR of 1.15 or greater at screening [8]. This change led to amyloid positive eligibility assessment outcomes for some individuals with a composite summary SUVR below the threshold (137 subjects, 11%, of the amyloid positive group included in these analyses). Amyloid positivity status for the analyses included here is based on the eligibility assessment outcomes.

### MRI acquisition and WMH quantification

**Fig. 1:**
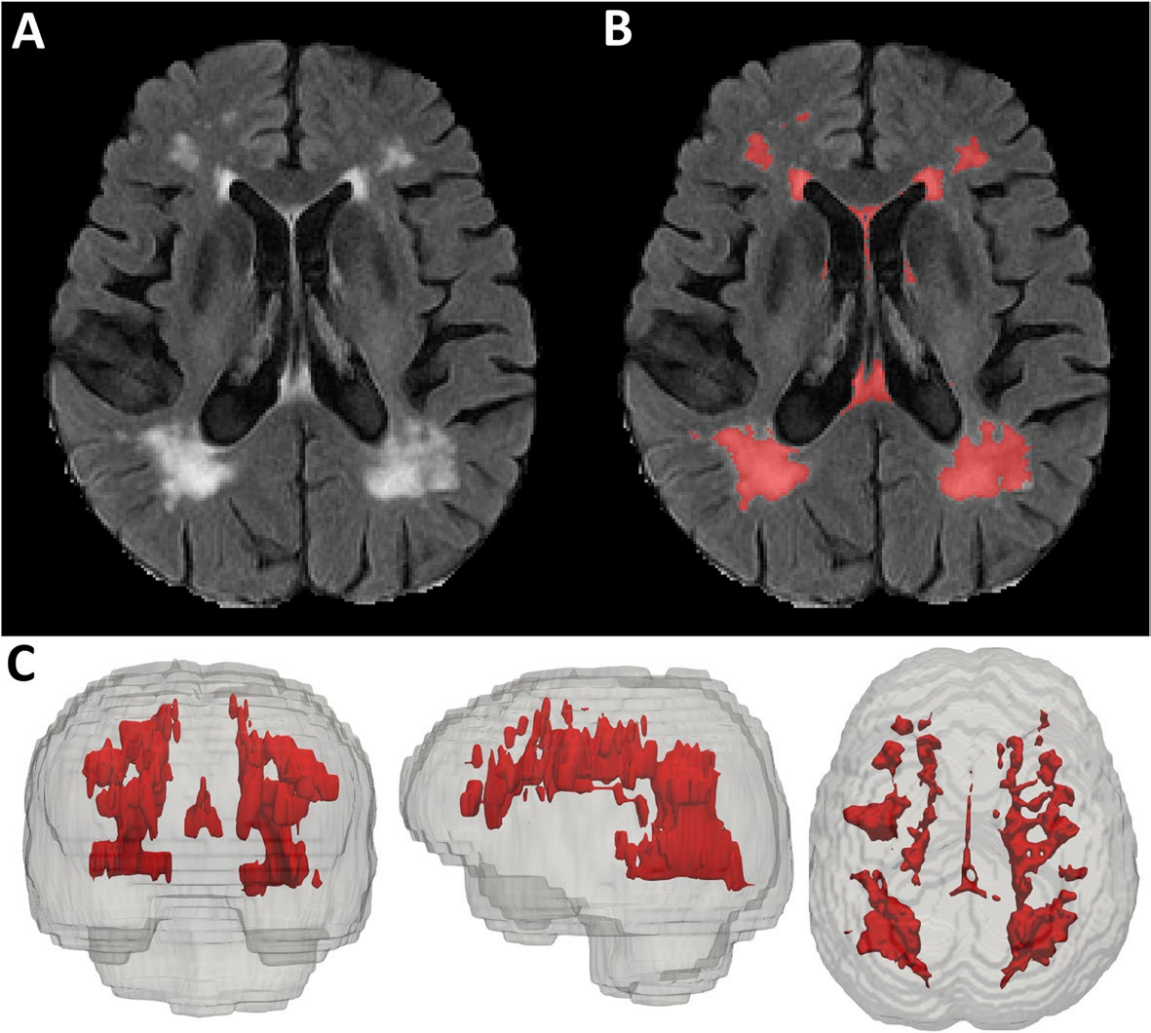
Example of a raw (**A**) and labeled for WMH (**B**) T2-weighted FLAIR image on a single axial slice from an A4 study participant. **C** 3D rendering of total WMH in same participant in coronal, sagittal, and axial view, respectively

Axial T2-weighted fluid-attenuated inversion recovery (FLAIR) MRI images (resolution, 0.86 mm, 0.86 mm, 5 mm; field of view, 256 × 256 × 35) were acquired for a majority of A4 and LEARN screening participants on study-approved 3T scanners with harmonized protocols after PET eligibility was determined. White matter hyperintensity volume was quantified with an automated algorithm with manual corrections (Fig. 1) [26]. Briefly, the method involves preprocessing the FLAIR images to remove non-brain tissue, correcting for intensity bias, and implementing a high-pass filter to remove voxels with intensity values equal to or below the mode. Intensity values of the preprocessed FLAIR images are then log transformed and fit to a half-Gaussian mixture model, in which the histogram containing an upper Gaussian curve captures image voxels within the distribution of WMH. The voxels within this range are labeled, and the mask is visually inspected and manually edited to remove any false-positive errors. We calculated total WMH volume and regional (frontal, temporal, parietal, and occipital lobe) WMH volumes in cm^3^. Regional WMH volumes were derived by co-registering a lobar atlas [27] to the FLAIR scans. Voxels within each of those regions are added and multiplied by the voxel dimensions to derive the regional WMH volumes.

### Statistical analysis

Demographic characteristics and total and regional WMH volumes were compared between participants in A4 and in LEARN with *t*-tests for continuous variables and chi-squared tests for proportional data. A series of general linear models tested the association of total and regional WMH volumes with PACC scores; these analyses included age, number of years of education, and amyloid status (elevated or not elevated) as additional predictor variables. In subsequent models, we added a term capturing the interaction between total or regional WMH and amyloid status to test whether the association of WMH volumes with PACC score differed in individuals with and without elevated amyloid. Akaike information criterion (AIC), a log-likelihood metric penalized for the number of model parameters (lower is better), was used to determine the most parsimonious model (i.e., model containing only main effects *versus* a model containing a WMH × amyloid interaction term). Adjusted R^2^ values, also penalized for the number of model parameters (higher is better), were used to compare the total variance in the outcome that was explained by the model. The square of the partial correlation was used to determine the proportion of the variance attributable to each covariate within the models. Note that because all participants included from the A4 trial had elevated amyloid levels and all participants in the LEARN study did not have elevated amyloid levels, main effects and interactions with amyloid status reflect the effects of substudy as well.

## Results

Table 1 displays the demographic characteristics of A4 and LEARN participants, corresponding to those with and without elevated amyloid levels, respectively. As is typical of clinical therapeutic trials in symptomatic or preclinical AD, participants were predominantly women, were in their early 70 s, were predominantly non-Latinx white, and had high levels of education. Participants in A4 (elevated amyloid levels) were older than those in LEARN (non-elevated amyloid) but were otherwise similar in sex/gender, race, ethnicity, and education. The two groups also did not differ in total WMH volumes; however, those enrolled in A4 had lower temporal lobe WMH volumes but greater occipital lobe WMH volumes.

**Fig. 2:**
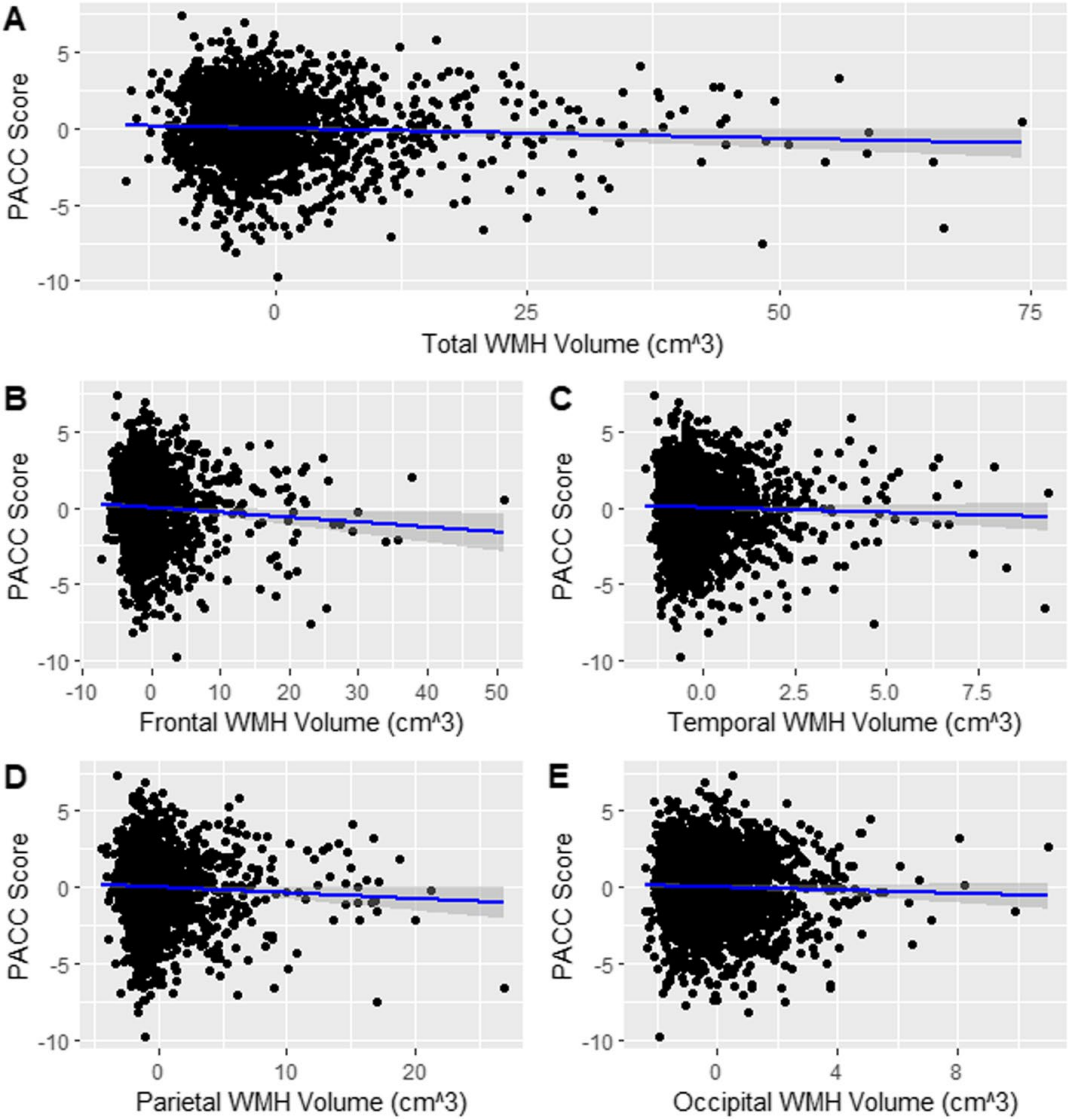
Scatterplots of total and regional WMH volumes against PACC scores. Plotted values have been residualized to account for covariates (age, education, and amyloid positivity status), leading to some negative values appearing on the *x*-axis

As seen in Table 2 and in Fig. 2, higher total, frontal, and parietal WMH volumes were associated with lower PACC scores. As expected, elevated amyloid was also associated with poorer PACC performance. The AIC, adjusted R^2^, and the interaction effect size (interaction effects =  − 0.0049–0.003, *p*-values = 0.48–0.95) converged to indicate that total and regional WMH volumes did not interact with amyloid status on PACC scores, suggesting that the association of total and regional WMH with cognitive outcomes is similar in those with and without elevated amyloid. The total variance accounted for in PACC scores was small to moderate for each model. In models with a significant WMH effects, total, frontal, and parietal WMH volumes accounted for 1.4%, 2.7%, and 1.6% of the total variance, respectively, while amyloid accounted for 3.5% in each model.

**Table 2:** The association of regional and total WMH volumes with PACC scores

	**Model 1 (frontal lobe)**			**Model 2 (temporal lobe)**			**Model 3 (parietal lobe)**			**Model 4 (occipital lobe)**			**Model 5 (total volume)**		
	**B**	**95% *CI***	***p*-value**	**B**	**95% *CI***	***p*-value**	**B**	**95% *CI***	***p*-value**	**B**	**95% *CI***	***p*-value**	**B**	**95% *CI***	***p*-value**
**Age**	− 0.17	[− 0.2, − 0.15]	*p* < 0.001	− 0.18	[− 0.20, − 0.16]	*p* < 0.001	− 0.18	[− 0.20, − 0.02]	*p* < 0.001	− 0.18	[− 0.20, − 0.16]	*p* < 0.001	− 0.18	[− 0.20, − 0.15]	*p* < 0.001
**Education**	0.12	[0.08, 0.17]	*p* < 0.001	0.13	[0.09, 0.17]	*p* < 0.001	0.13	[0.08, 0.17]	*p* < 0.001	0.13	[0.09, 0.17]	*p* < 0.001	0.13	[0.09, 0.17]	*p* < 0.001
**WMH**^a^	− 0.03	[− 0.06, − 0.01]	*p* = 0.009	− 0.07	[− 0.17, 0.03]	*p* = 0.15	− 0.04	[− 0.08, − 0.000008]	*p* = 0.04	− 0.05	[− 0.13, 0.03]	*p* = 0.20	− 0.01	[− 0.03, − 0.001]	*p* = 0.03
**Amyloid**	− 0.38	[− 0.62, − 0.13]	*p* = 0.003	− 0.38	[− 0.63, − 0.13]	*p* = 0.002	− 0.38	[− 0.62, − 0.13]	*p* = 0.003	− 0.37	[− 0.62, − 0.12]	*p* = 0.003	− 0.38	[− 0.62, − 0.13]	*p* = 0.003
**Adjusted R**^**2**^	0.141			0.138			0.139			0.138			0.140		
**ΔR**^**2**^** with inclusion of interaction term**	0.0005			0.0005			0.0005			0.0003			0.0005		
**ΔAIC with inclusion of interaction term**	3.54			1.44			3.35			1.09			2.01		

Five separate models were run, which included regional (frontal, temporal, parietal, and occipital) and total WMH volumes, respectively, as the primary WMH predictor of PACC, together with age, education, and amyloid status, which were invariant across models. Included statistics reflect unstandardized B estimate, 95% confidence intervals, and *p*-values of the association of each factor (age, education, regional or total WMH, and amyloid SUVR) with PACC scores. The bottom two rows show change in adjusted *R*^2^ and AIC when comparing models with and without a WMH by amyloid interaction term. A positive delta AIC indicates that there is less parsimony with interaction term in the model

^a^The “WMH” variable refers to frontal, temporal, parietal, occipital, and total WMH volume for each of the five models, respectively

## Discussion

We found that regionally distributed WMH, an indicator of small vessel cerebrovascular disease, are associated with cognitive functioning in a group of participants being considered for enrollment in a secondary prevention trial for AD targeting beta amyloid. As expected, the PET biomarker reflecting beta amyloid pathology was strongly related to cognition, but WMH additionally contributed independently and significantly to the amount of variance in the cognitive outcome. The findings have important implications for our understanding of sources of cognitive impairment in preclinical AD, for strategies to analyze and interpret AD clinical trial data, and for planning of future prevention or intervention trials in AD.

It is now well-established that regional cerebrovascular disease is associated with cognition, cognitive decline, and risk of and progression of both late onset and autosomal dominant AD [15–18]. This observation is recapitulated in the current study, which included older adults considered for a secondary prevention trial. We confirmed an association of total WMH with cognitive outcomes. In terms of impact of regional WMH distribution, we previously showed consistent associations between posterior distribution, typically parietal lobe, WMH, and outcomes related to AD [28]. Here, we observed the same regional effect but also found that frontal lobe WMH, which are typically attributed to age- and risk factor-related ischemic changes [29], are also related to cognitive outcomes. It is unclear what factors mediate the regional distribution of AD-associated WMH, but it is interesting to note that several pathophysiology features of AD converge in posterior areas relatively early in the disease process, including cerebral microbleeds, amyloid and tau pathology, atrophy, and glucose hypometabolism [30–33]. We speculate that upstream blood flow abnormalities in posterior regions may mediate some of this convergence. As in previous studies [34, 35], WMH volume was independent of and did not interact with amyloid status in its relationship with cognitive outcomes. Some work suggests that amyloid pathology does interact with small vessel cerebrovascular disease to affect rates of cognitive decline [36], so despite the independent effects we observed of the two markers cross-sectionally, they may have a synergistic impact on change in cognitive outcomes. Additionally, our previous work demonstrates an effect of small vessel cerebrovascular disease on tau pathology [37], raising the possibility of direct downstream consequence of cerebrovascular disease on AD pathophysiology. The findings in the current study confirm that even in typical prospective AD clinical trial participants, cerebrovascular disease contributes to the clinical profile. Notably, the impact of cerebrovascular disease on cognition was not trivial; whereas amyloid positivity status accounted for 3.5% variance in PACC scores, frontal lobe WMH accounted for 2.7%.

The relationship between regional WMH volume and performance on the PACC, which was the primary clinical efficacy outcome of the trial, suggests that WMH should be considered explicitly in the design and analysis of clinical trial data. We are unaware of trials that have examined baseline WMH as potential effect modifiers or factors that would need statistical adjustment when considering efficacy. However, the relationship we observed indicates WMH as a source of variance in trial outcomes that could potentially obscure treatment effects. The results of the A4 trial showed that solanezumab did not slow cognitive decline compared with placebo [34]; an important post hoc analysis should examine whether WMH affected the efficacy outcomes in this trial. Similarly, it is now well established that brain edema and hemorrhage, the so-called amyloid-related imaging abnormalities (ARIA-E and ARIA-H, respectively), are common side effects of monoclonal antibody treatments that target amyloid [38]. The presence of baseline microbleeds is one predictor of these adverse outcomes [38], but future analyses should consider whether small vessel disease manifesting as WMH may also signal who might be at risk for adverse events. Cerebral microbleeds may be difficult to detect, and their visualization is dependent on MRI scan protocols, resolution, and operator expertise; on the other hand, WMH are easily visualized and quantifiable and may provide similar or better indicators of risk. Future trials should consider analyses involving WMH for both efficacy and safety outcomes.

### Limitations

There is considerable debate about whether WMH in the context of AD represent a purely vascular phenomenon or whether they are consequences of AD-related neurodegeneration [39, 40]. We have argued against the latter based on animal studies, anatomical distribution, temporality, and dissociations between vascular risk factors and WMH severity [41]. In the A4 trial, which includes individuals who are presymptomatic or have very mild symptoms, we would also not expect there to be significant white matter degeneration secondary to AD pathophysiology at such early disease stages. Additionally, while the A4 trial was designed to be more inclusive of race and ethnicity groups than previous AD trials [8], there was still bias [42], and the target numbers for inclusion of enrolled participants from racially and ethnically minoritized backgrounds were not reached. We [43, 44] and others [45] have documented differences in WMH volume across race/ethnicity minoritized groups and in the impact of WMH on cognitive outcomes. As recruitment and enrollment strategies of AD trials continue to improve with respect to more inclusive participation, the potential impact of cerebrovascular disease on the efficacy, and possibly safety, of trial outcomes will become more relevant.

## Conclusions

Given the multifactorial etiology of cognitive decline in AD, efforts towards risk reduction and prevention via multidomain interventions that include management of cerebrovascular disease [46, 47], combination therapies [48], or explicit inclusion of individuals with cerebrovascular disease will continue to emerge as important strategies. Our study confirms the importance of multiple etiological factors in cognitive outcomes even among individuals considered for single mechanism secondary prevention AD trials.

## Data Availability

A4 trial data are available through the *Laboratory of Neuroimaging, Image & Data Archive*.

## Abbreviations

A4: Anti-amyloid in asymptomatic alzheimer’s disease
AD: Alzheimer’s disease
AIC: Akaike information criterion
FLAIR: Fluid-attenuated inversion recovery
LEARN: Longitudinal evaluation of amyloid risk and neurodegeneration
LMDR: Logical memory delayed recall
MCI: Mild cognitive impairment
MMSE: Mini-mental state examination
MRI: Magnetic resonance imaging
PACC: Preclinical Alzheimer cognitive composite
PET: Positron emission tomography
SUVR: Standardized uptake value ratio
WMH: White matter hyperintensities
